# Evaluation of analgesic effects of intrathecal clonidine along with bupivacaine in cesarean section

**DOI:** 10.4103/1658-354X.76499

**Published:** 2011

**Authors:** Nikhil Kothari, Jaishri Bogra, Ajay K. Chaudhary

**Affiliations:** *Department of Anesthesiology, CSMMU (erstwhile KGMC), Lucknow, Uttar Pradesh, India*

**Keywords:** *Cesarean section*, *intrathecal clonidine*, *bupivacaine*

## Abstract

**Aims and Context:**

The objective of the present study was to evaluate the analgesic and adverse effects of intrathecal clonidine with hyperbaric bupivacaine in spinal anesthesia.

**Settings and Design:**

Randomized single blind trial.

**Methods:**

210 ASA I-II pregnant females undergoing emergency cesarean section were randomized in a single-blind fashion to one of the three groups. In group I (n=70) patients received 12.5 mg of 0.5% hyperbaric bupivacaine intrathecally. In group II (n=70) patients received intrathecal mixture of 0.5% hyperbaric bupivacaine (8 mg) and clonidine 50 *μ*g. In group III (n=70), patients received 0.5% hyperbaric bupivacaine (10 mg) intrathecally along with 50 *μ*g of clonidine.

**Statistical Analysis Used:**

Groups were compared using one-way ANOVA with the Bonferroni multiple comparison post hoc test. The proportion of adverse events was compared using the chi-square test (χ^2^ =57.2410).

**Results:**

On adding 50 *μ*g clonidine, we were able to reduce intrathecal dose of bupivacaine for cesarean section to 8 mg. Patients receiving intrathecal clonidine along with bupivacaine had significantly long lasting analgesia with lower bupivacaine dose [246.21±5.15 min. (group II) vs 146.0±4.55 min (group I), *P*=0.021; 95% confidence interval: 238.01-257.40, group II and 134.99-157.0 group I].

**Conclusions:**

Addition of intrathecal clonidine causes some sedation in the postoperative period, but it provides adequate analgesia and motor paralysis at lower dose of bupivacaine. It also significantly prolongs postoperative pain relief.

## INTRODUCTION

Spinal anesthesia has become the preferred anesthesia for cesarean section. Spinal anesthesia is simple to perform, economical, produces rapid onset of anesthesia and complete muscle relaxation. Although prolongation of the effects of local anesthetics has been reported for oral and intravenous clonidine, the intrathecal route is more effective; clonidine prolongs the duration of action of intrathecally administered local anesthetics and has potent antinociceptive properties.[1] A recent report established 1 μg/kg intrathecal clonidine as an adequate dose for prolonging bupivacaine spinal anesthesia in pregnant females.[2] It was observed that intrathecal clonidine upto 150 μg prolongs spinal anesthesia and analgesia but hemodynamic instability was seen with higher doses,[3] so intrathecal doses of clonidine were titrated to 50 μg for pregnant females in our study in order to achieve adequate analgesia along with hemodynamic stability.

The primary outcomes studied were time to regression of spinal blockade below level L1 and duration of pain relief, defined as the time from intrathecal clonidine administration to first request for supplemental analgesia by patients. Postoperative cumulative analgesic consumption, visual analog scale (VAS) pain scores, motor blockade, hemodynamic variables, use of intravenous ephedrine, additional fluid requirements, and sedation were also recorded.

## METHODS

A randomized single blind trial was performed on 210 parturients undergoing emergency cesarean section under spinal anesthesia. Patients were randomized into three groups (n=70 in each group) using computer generated random number table. These groups were further classified on the basis of intrathecal drug combination used.

**Table:** 

Group I:	12.5 mg 0.5% hyperbaric bupivacaine (Anawin, Neon labs).
Group II:	8 mg 0.5% hyperbaric bupivacaine along with 50 μg clonidine
Group III:	10 mg 0.5% hyperbaric bupivacaine along with 50 μg clonidine.

Patients were explained about the intrathecal use of new drug and written informed consent was taken, patients were given full liberty to opt for conventional methods of anesthesia. Patients included in the study were not aware of the drug combination which they received for spinal anesthesia in order to rule out the bias regarding use of rescue analgesia in the postoperative period. Patients included for this study belonged to American Society of Anesthesiology (ASA) grade I or II, having full-term normal pregnancy. None of the patients had any contraindication for spinal anesthesia. Exclusion criteria were complicated pregnancies such as multiple pregnancy, pregnancy-induced hypertension, placenta previa and antenatal patients with acute fetal distress, patients with previous abdominal surgeries and patients having body weight>80 kg.

All the parturients were preloaded with 0.5 to 1.0 L of ringer lactate via 18 gauge venous catheter. Base line parameters for pulse rate, systolic and diastolic blood pressures were recorded in all three groups and were found to be statistically insignificant. In group I (n=70), we tried to find out optimal dose of intrathecal bupivacaine (12.5 mg) which was not associated with visceral pain.[4] Visceral pain was assessed on the basis of previously explained visual analog score, judged by the patient at the time of uterine incision and peritoneal cleaning. In group II (n=70), intrathecal clonidine (50μgm) was added and dose of bupivacaine was reduced to 8 mg.[5] The second group was made with an idea to find out the low dose bupivacaine-clonidine combination that was not associated with visceral pain. In the third group (n=70), parturients were given bupivacaine 10 mg intrathecally along with 50 μg clonidine. Spinal anesthesia was given in lateral position between L_2_ and L_3_ vertebrae using 25 gauge Whitacre needle taking complete aseptic precautions. Immediately after the block, each parturient was placed with a wedge under righthip.[6] Pulse and non-invasive blood pressure were measured every 5 min for first 30 min and thereafter every 10 min. Hypotension was defined as 20% decrease from baseline MAP. It was treated with i.v. fluid bolus and with 3 mg incremental boluses of i.v. ephedrine. Total ephedrine requirements, number and duration of hypotension episodes were recorded. Patients breathed spontaneously and supplementary oxygen was given through a facemask during the operation. Urine output was also monitored. Sensory block was tested by cold, touch, and pinprick along the midclavicular line till the block reached T6 level and then the surgical incision was allowed.[7] The quality of anesthesia (judged by anesthetists), the quality of muscle relaxation (judged by surgeon) and degree of intraoperative comfort (judged by patient) were recorded as excellent, good, fair, or poor. Intraoperative pain and the feeling of discomfort were evaluated by the patient and recorded by an observer unaware of patient data using visual analog scale. Development of motor block was assessed with the Bromage Scale (0=no motor block to 3=complete motor block of both lower limbs). Incidence of nausea, vomiting, itching, shivering, pruritus, and sedation during operation; the time required for sensory recovery below L1 dermatome, motor recovery (ability to move lower limbs), and onset of postoperative pain were recorded. Apgar score of all the babies at 1, 5, and10 min and maternal respiratory depression were recorded.[8]

### Statistical analysis

Three groups were compared using one-way ANOVA with the Bonferroni multiple comparison *post hoc* test. Data were considered significant with *P*<0.05. The proportion of adverse events was compared using the chi-square test (χ^2^=57.24, 10) and statistical significance was observed at *P*<0.001.

## RESULTS

All three groups were almost similar with respect to age, height, and weight [Table 1]. The onset of sensory block occurs faster with higher bupivacaine doses [Table 2] as compared to bupivacaine-clonidine combination groups (gr I>gr II>gr III). It was observed that parturients in all the groups had complete motor block (Bromage scale).

**Table 1:** Patient characteristics (Mean ± SD)

	Group I	Group II	Group III	*P*
Age (years)	26±2	26±3	27±3	NS
Height (cm)	156.5±2.8	157.4±3.0	157.5±3.1	NS
Weight (kg)	63.5±4.3	61.5±4.1	64.2±4.4	NS

**Table 2:** Onset of sensory block up to T_6_ (time in minutes)

	Mean+SE	N	95% CI	
Group I	5.5+0.66	70	4.83 to 6.16	
Group II	6.8+0.26	70	6.53 to 7.06	
Group III	5.6+0.28	70	5.31 to 5.88	

*P*=0.0073, groups I and II.

**Figure 1:**
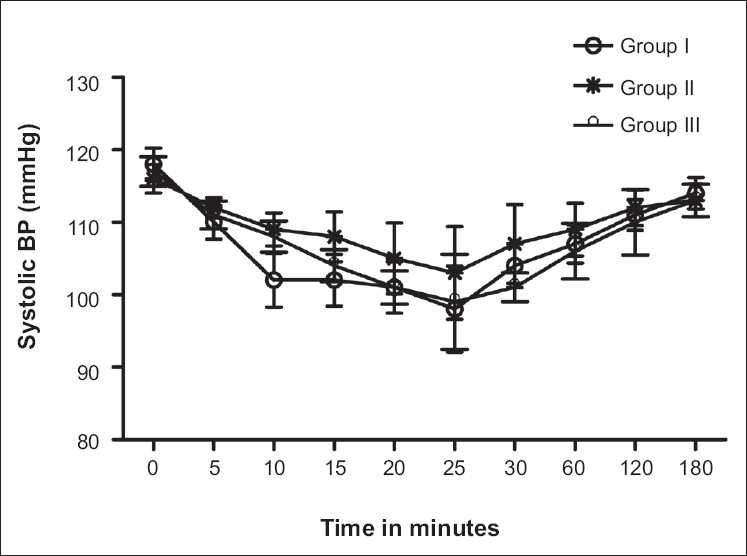
Changes in systolic blood pressure with respect to time, after giving spinal anesthesia

Incidence of intraoperative visceral pain (judged by patient) was significantly reduced after adding clonidine with lower bupivacaine dose; visceral pain was seen in only three cases in group II, which was managed by i.v. paracetamol infusion (1 g, Perfalgan). No incidence of visceral pain was observed in groups II and I. In our study the maximum fall in systolic blood pressure from baseline was observed at 25 min [Figure 1]. In bupivacaine-clonidine combination groups, fall in systolic blood pressure from baseline increased with increasing bupivacaine dose (gr III>gr II). On comparing hemodynamic stability we found group II was hemodynamically more stable than group I as lower dose of bupivacaine was used. Incidence of hypotension (20% decrease from baseline MAP) increases with higher dose of bupivacaine [Figure 1]. We also observed statistically significant difference in incidence of bradycardia between groups I and II.

**Figure 2:**
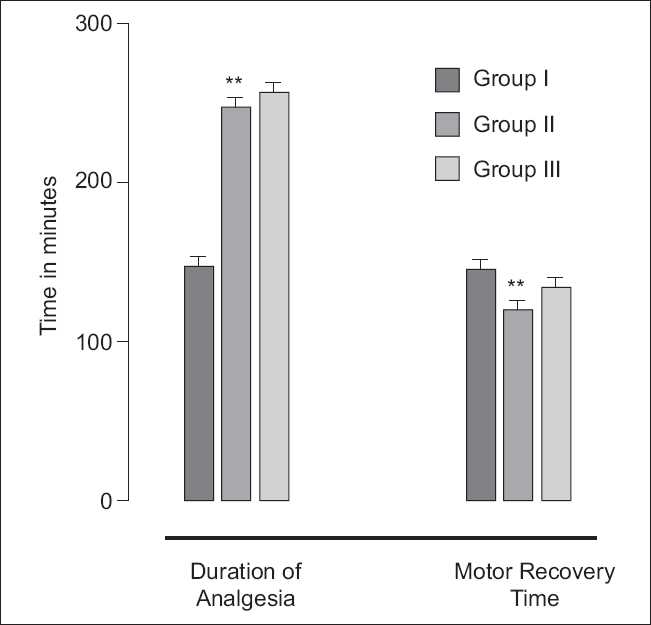
Duration of analgesia and motor paralysis

Though increasing doses of bupivacaine, duration of post-operative analgesia was prolonged but with addition of clonidine to bupivacaine, longer post-operative analgesia occurs at lesser intrathecal dose of bupivacaine [246.21±5.15 min. (group II) *vs* 146.0±4.55 min (group I), *P*=0.021; 95% confidence interval: 238.01-257.40, group II and 134.99-157.0 group I; [Figure 2]. Motor recovery takes longer time with increasing doses of bupivacaine (grI>gr III>gr II).

We found no sedation in group I. Sedation was observed in 36-45% parturients of bupivacaine-clonidine combination groups, who were drowsy [Table 3]. Statistically significant difference exists in sedation caused by addition of intrathecal clonidine to bupivacaine (*P*<0.001). Significantly lower incidence of nausea in bupivacaine-clonidine combination groups as compared with bupivacaine only groups [Table 3]. Lower incidence of shivering (1 to 5%) was present in parturients with bupivacaine-clonidine combination but not statistically significant when compared with bupivacaine only group (7%). Apgar Score of babies (at 1, 5 and 10 minutes) was unaffected when 50*μ*g intrathecal Clonidine used in cesarean section.

**Table 3:** Incidence of side effects

(n=70)	Group I		Group II		Group III	
	No.	%	No.	%	No.	%
Hypotension	50	71.42	25	35.71	40	57.14
Bradycardia*	16	22.85	5	7.14	10	14.28
Sedation**	2	2.85	36	51.42	45	64.28
Nausea	20	28.57	5	7.14	13	18.57
Vomit	5	7.14	3	4.28	2	2.85
Rigors	5	7.14	1	1.42	4	5.71

χ2=57.24, 10

* *P*<0.01

** *P*<0.001

## DISCUSSION

**Figure 3:**
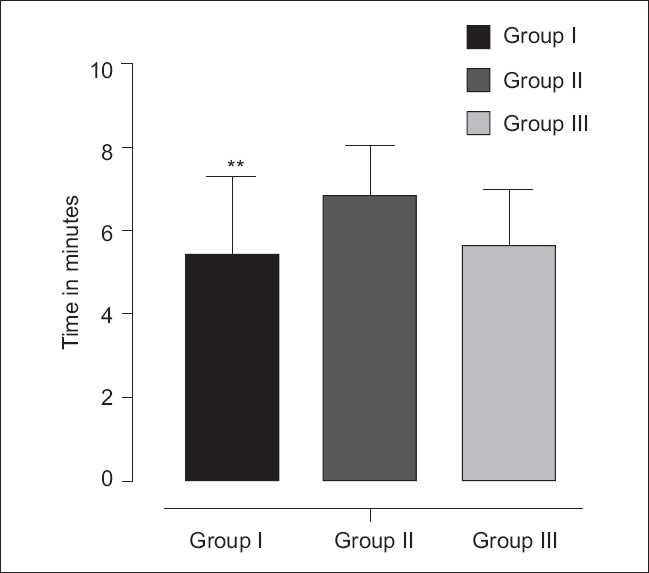
Time for onset of sensory block

Recent trends of obstetric anesthesia show increased popularity of regional anesthesia among obstetric anesthetists. General anesthesia in the cesarean section is associated with higher mortality rate in comparison to regional anesthesia. Regional anesthesia is also having its own demerits. Deaths in regional anesthesia are primarily related to excessively high regional blocks and toxicity of local anesthetics. Reduction in doses and improvement in the technique to avoid higher block levels and heightened awareness of toxicity of local anesthetics have contributed to reduction of complications related to regionalanesthesia.[9] On comparing progression of sensory block to T6 dermatome_;_ in our study we found the mean time duration of 6.8 min in group II and minimum time of 5.50 min in group I [Figure 3].

In this study muscle relaxation was found to be excellent in all three groups (judged by surgeon). This is supported by Ogun *et al*.[10] who also reported excellent muscle relaxation with or without addition of 75 μg clonidine to 8 and10 mg 0.5% hyperbaric bupivacaine in the cesarean section. Visceral pain is a common problem in the cesarean section under spinal anesthesia. It is a poorly localized type of pain that appears to come from deep inside the body. It is often associated with autonomic activity causing nausea, vomiting, sweating, and changes in blood pressure and heart rate. Visceral pain in the cesarean section is experienced when the uterus is exteriorized and peritoneum is closed (judged by patient). In our study we found no visceral pain in group I. However visceral pain incidence could be reduced with lower bupivacaine doses of 8 mg by adding 50 μg of clonidine. In our study we found the depth of anesthesia in group II to be equivalent to group III, though the dose of bupivacaine was less. This is observed that by addition of clonidine, adequate depth of spinal anesthesia can be achieved at much lower doses of bupivacaine.[11]

**Figure 4:**
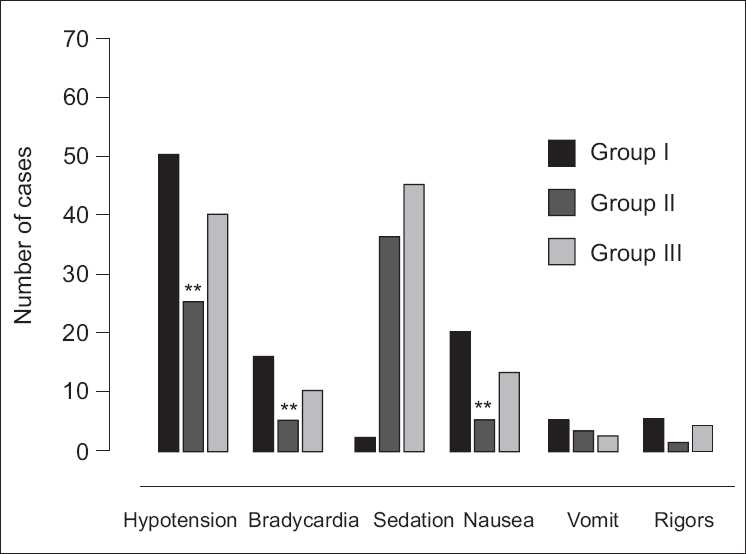
Adverse events (number of episodes)

Bradycardia in spinal anesthesia is believed to result from at least two causes: blockade of sympathetic cardio accelerator fibers and decreased venous return to the heart. Sympathetic cardio accelerator fibers arise from T_1_ to T_4_, so that a sympathetic block height to T_1_ should completely eliminate sympathetic flow to the heart. However, this level of sympathetic block is associated with peripheral vasodilatation and a reduction in preload. The decrease is preload is believed to be the most important cause of decrease in heart rate. Bradycardia in our study was found in 31 out of 210 parturients [Figure 4]. Higher incidence was observed with higher bupivacaine doses. There was no difference in incidence of bradycardia by addition of clonidine in groups II and III.

By addition of 50 *μ*g intrathecal clonidine we found that 35-45% of the patients were drowsy (assessed by Ramsay sedation scale) as compared to those without clonidine addition [Table 3]. Intrathecal clonidine increases sedation but patients were responsive to simple verbal commands. In our study we found increased incidence of nausea and vomiting with higher bupivacaine dose [Table 3] but no significant increase in side effects by adding clonidine. Our study showed that by adding clonidine duration of analgesia was prolonged but the side effects were less as dose of bupivacaine was reduced.[12] Apgar scores of neonate (at 1, 5, and 10 min) were not impaired by addition of 50 μg intrathecal clonidine to 0.5% hyperbaric bupivacaine.

Motor recovery was not affected by addition of 50 μg intrathecal clonidine.[13] Our study shows that motor recovery is more dependent on dose of bupivacaine rather than concentration of clonidine; motor recovery is longer with increasing dose of bupivacaine but by adding clonidine to intrathecal bupivacaine, duration of motor blockade was not prolonged.[14]

## CONCLUSION

Addition of intrathecal clonidine provides adequate analgesia and motor paralysis at significantly lower dose of bupivacaine. It causes some sedation in the postoperative period but patients were responsive to simple verbal commands, thus intrathecal use of clonidine is recommended to reduce dose of bupivacaine for better hemodynamic stability and prolonged postoperative analgesia.
